# An Eye-Tracking System based on Inner Corner-Pupil Center Vector and Deep Neural Network

**DOI:** 10.3390/s20010025

**Published:** 2019-12-19

**Authors:** Mu-Chun Su, Tat-Meng U, Yi-Zeng Hsieh, Zhe-Fu Yeh, Shu-Fang Lee, Shih-Syun Lin

**Affiliations:** 1 Department of Computer Science and Information Engineering, National Central University, Taoyuan City 32001, Taiwan; muchun@csie.ncu.edu.tw (M.-C.S.); chrisu1023@gmail.com (T.-M.U); 2 Department of Electrical Engineering, National Taiwan Ocean University, Keelung City 20224, Taiwan; 3 Institute of Food Safety and Risk Management, National Taiwan Ocean University, Keelung City 20224, Taiwan; 4 Center of Excellence for Ocean Engineering, National Taiwan Ocean University, Keelung City 20224, Taiwan; 5 Landseed International Hospital, Taoyuan City 324, Taiwan; arckse123@hotmail.com (Z.-F.Y.); leesf@landseed.com.tw (S.-F.L.); 6 Department of Computer Science and Engineering, National Taiwan Ocean University, Keelung City 20224, Taiwan

**Keywords:** eye tracking, deep neural network, inner corner-pupil center vector

## Abstract

The human eye is a vital sensory organ that provides us with visual information about the world around us. It can also convey such information as our emotional state to people with whom we interact. In technology, eye tracking has become a hot research topic recently, and a growing number of eye-tracking devices have been widely applied in fields such as psychology, medicine, education, and virtual reality. However, most commercially available eye trackers are prohibitively expensive and require that the user’s head remain completely stationary in order to accurately estimate the direction of their gaze. To address these drawbacks, this paper proposes an inner corner-pupil center vector (ICPCV) eye-tracking system based on a deep neural network, which does not require that the user’s head remain stationary or expensive hardware to operate. The performance of the proposed system is compared with those of other currently available eye-tracking estimation algorithms, and the results show that it outperforms these systems.

## 1. Introduction

The human eye is a vital sensory organ that receives external visual information about the world around us. However, it can also convey emotion-related information, such as by the direction of the gaze or how wide the eyelids open or close, as well as imply how we experience the world, to some degree (environmental brightness, for example). Eye-tracking has thus become a hot research topic as technological developments have enabled more accurate measurement of various vectors. Such eye-tracking technology is extremely valuable in many fields. For example, people with disabilities, such as partial paralysis, but who are still able to move their eyes, can use eye tracking-based systems to communicate and interact with computers and even robotic devices and are thus afforded increasingly more comprehensive methods of interacting with their environment and communication with every new advance in eye-tracking technology. Thus eye-tracking technology is highly sought-after in the medical field. In recent years, eye tracking has been applied in a much wider variety of fields, especially in virtual reality, allowing users to wear head-mounted devices. These devices increase the immersive nature of the virtual space by using eye-tracking to create visual focus. In the rapidly growing field of digital learning, some experts have proposed using eye-tracking to determine what learners most focus on during the digital learning process.

Eye-tracking has great potential for development, and eye-tracking technology will soon be available in everyday life. Currently, available eye-tracking products are expensive, however, and require that users’ heads remain stationary. This study, therefore, aims to use a web camera (webcam) in conjunction with a deep neural network to measure a user’s point of gaze coordinates on the screen of an eye-tracking system.

In the proposed system, the webcam captures the features of the face and eyes as the user looks at the correct points on the screen, and the available feature information is then calculated. This study then uses these features in combination with neural network models to train an eye-tracking system that can estimate the user’s point of gaze. This study achieves the following objectives and contributions:

- When a user looks at the screen, the system can accurately estimate the point of gaze coordinates.
- The proposed system does not require a fixed head apparatus, and can still accurately estimate the point of gaze when the users move their head.
- Our system is a low-cost eye-tracking system that can just run on the user’s PC and webcam, without the need for other commercial equipment.
- Our system can be easy for users to operate or set up the system and more comfortable for some disabled users.

## 2. Related Works

Eye-tracking refers to the tracking of eye movements by measuring the gaze direction or gaze point of the user, by using hardware devices and algorithms. An eye tracker is a hardware device that measures eye movement information.

The application analysis of eye-tracking is currently divided into two categories: eye movement trajectories, and hot maps. Eye movement trajectory may be analyzed when users move their eyes to view an object, while hot maps analyze how a user looks at an object over a period of time. For example, when a user browses a shopping website, eye tracking can be used to identify the area that is of most interest to the user. Early optical eye-tracking studies predominantly used head-mounted devices to capture eye and screen information [1,2]. Head-mounted devices are available commercially today as wearable eye-tracking devices. As eye tracking is being used more widely in commercial applications, these wearable devices have become more lightweight, with such models as SMI Eye-Tracking Glasses [3] and Tobii Pro Glasses 2 [4].

In addition to head-mounted eye-tracking devices, remote eye trackers, such as the Tribe [5] and Tobii Eye Tracker Pro X3-120 [6] have also been developed. Most telemetry eye trackers use infrared light to capture image information. However, the price of most eye trackers is around 30 million Taiwan dollars [7], greatly beyond the means of the general public. Therefore, methods of making such devices more efficient, and thus requiring less expensive hardware, have been the focus of a number of recent studies. Zhang et al. [8] proposed gathering the dynamic areas of interest (AOI) and combining them with eye movement data. The study [9] focused on capabilities for quantitative, statistical, and visual analysis of eye gaze data, as well as the generation of static and dynamic visual stimuli for sample gaze data collection. Kurzhals et al. [10] demonstrated their approach with eye-tracking data from a real experiment, and compared it to an analysis of the data by manual annotation of dynamic AOI. Zhang and Yuan [11] proposed the assessment of advert element-related eye movement behaviors in order to predict the traditional high-order advertising effectiveness for video advertising. The research [12] focused on determining the effects of data density, display organization, and stress on visual search performance and associated eye movements (obtained via eye-tracking). Yan et al. [13] proposed an eye-tracking technology to record and analyze a user’s eye movement during a test, in order to infer a user’s psychological and cognitive state. Wu et al. [14] proposed a system based on Kinect 2.0 to improve life quality for people with upper limb disabilities. With recent advances in deep learning, many new methods based on convolutional neural networks (CNNs) have been proposed, and have achieved good performances on salient benchmarks [15,16]. For gaze detection on a 2D screen [17,18], the screen used for detecting the gaze is placed in a fixed location. Such methods are certainly useful for HCI (human–computer interaction). Ni and Sun [19] proposed leveraging deep learning theory to develop a remote binocular vision system for pupil diameter estimation. Our proposed eye-tracking system is not mounted on the device on the head, and the user can feel free to the human-machine interface design without the mounted the head device. Also, nowadays the commercial eye-tracking device is high cost and the user must be trained to operate the eye-tracking system. Fortunately, our proposed system is low cost and the low-cost RGB camera or webcam can easily be built to integrate into our system. The user adopting our system can easily learn to operate the eye-tracking action.

## 3. The Proposed Method

**Figure 1:**
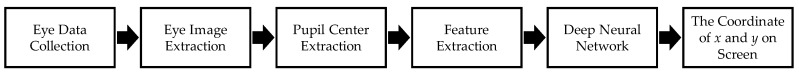
The workflow of our system.

In order to train a neural network, the information collection phase first retrieves the feature information, and then the required characteristic values are calculated. Any information containing errors is filtered out, and the remaining data are then used to train the neural network model. Figure 1 shows the workflow of our proposed system.

### 3.1. Data Collection

**Figure 2:**
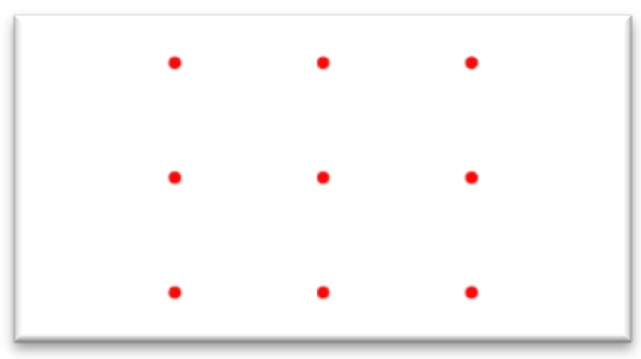
The calibration point map.

In order to train neural networks, training resources must first be collected. These are collected in two ways. The first is to use a head holder fixing the position of the user’s head ( the experiment in this paper places the user’s face at a distance of 40 cm from the screen) while the camera takes pictures focusing on the middle of the positions. The second collection method does not limit the position of the user’s head, which can move freely while data are collected. The training data collection method employs nine-point calibration, points on the screen, as shown in Figure 2. The users focus on each point in sequence, with about 1.5 s intervals in between, allowing them to focus on the correct point. Each correct point sampling is made up of 40 frames, so there will be a total of 360 calibration data.

**Figure 3:**
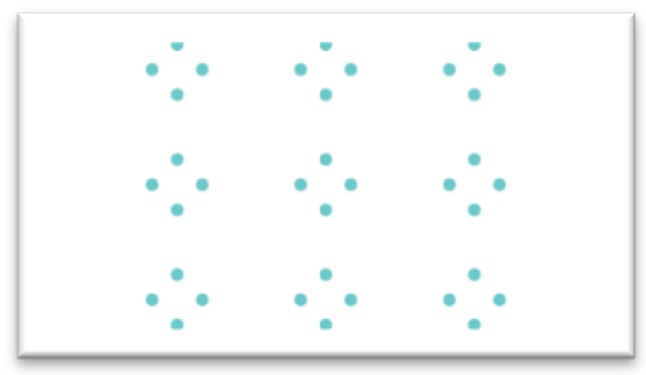
The test data collection.

In order to collect test data, this study uses the point distribution shown in Figure 3. The point positions are collected as 9 calibration points made up of 80 pixels, in sequence from top to bottom, left to right, totaling 36 calibration points. As with the training data collection, the user focuses on each calibration point in sequence, yielding a total of 360 data.

### 3.2. Eye Image Extraction

Our system will first detect the eyes existing or not. If the eyes are not found, the system will stop all action until the eyes will be found. This instruction will control the system to avoid the eyes disappearing.

This study captures images of the user’s eyes using the webcam image and the Haar feature-based cascade classifier. However, this method may not completely and accurately capture the eye images, so this study also adopts the concept of region of interest (ROI). In the field of computer vision, ROI refers to a specific area in a complete image; calculating this area can reduce processing time and increase accuracy.

**Figure 4:**
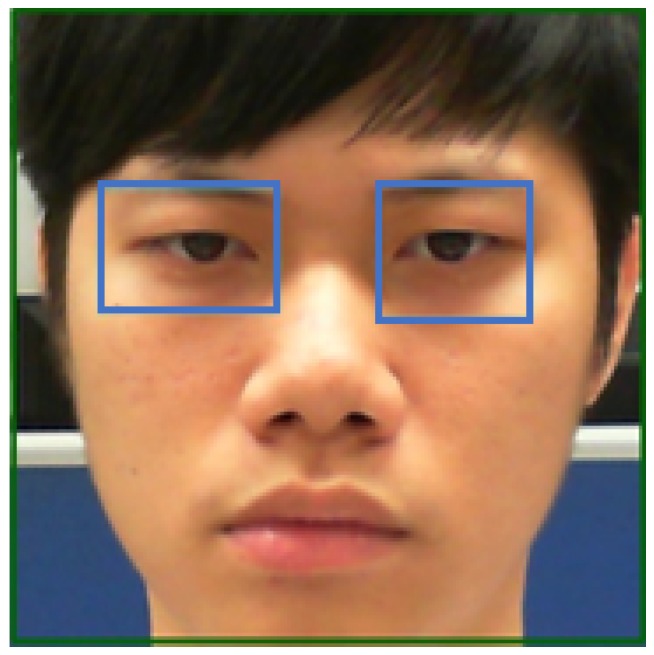
Eye regions.

In this paper, the image of the user’s face is divided into four regions, with the eyes falling within the second region from the top of the image. The first two zones are for the eye ROI, followed by the ROI detection in the left and right eyes. This paper divides the successfully captured face image vertically into five regions. Of these, the left eye falls within the second region, and the right eye falls within the fourth region, as shown in Figure 4.

### 3.3. Pupil Center Extraction

**Figure 5:**
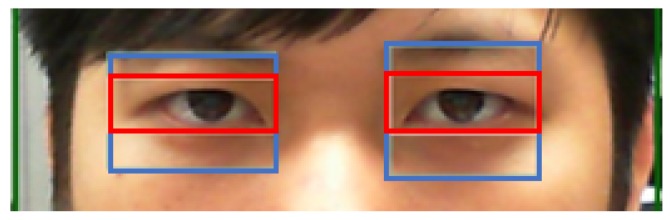
The red region is the ROI after removing the eyebrows.

If the eye region is successfully captured, the eyebrows are then excluded from the captured image region, so that the pupils can be accurately identified. Therefore, this study divides the captured eye images into 3 regions. The second region is the ROI. The eyebrows can then be cropped from the image, and the image can be more accurately processed, with the focus on the ROI, as shown in Figure 5.

In order to extract the eye image converted to HSV and extract the Value channel, this study binarizes the grayscale image of the Value channel. The resulting black area is the pupil. By filtering out incomplete data or noise, morphological image processing such as erosion and dilation can be used.

**Figure 6:**
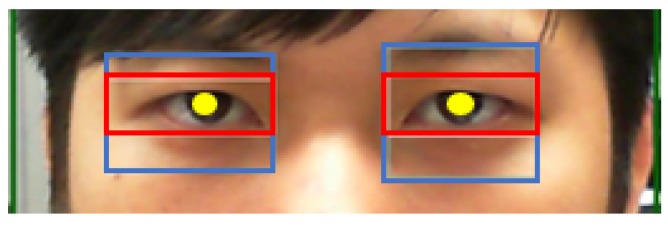
The position of the pupil center.

To estimate the center of the pupil, this study calculates the center of gravity by processing the black portion of the image, using Equations (1) and (2), respectively, to calculate the *x* and *y* coordinates of the center of gravity. This allows the estimation of the position of the center of the pupil $\left(C_{x},C_{y}\right)$, as shown in Figure 6:

(1) $$C_{x}=\frac{\sum xB_{x}}{\sum B_{x}},$$

(2) $$C_{y}=\frac{\sum yB_{y}}{\sum B_{y}}.$$

### 3.4. Capturing Eye Corners

**Figure 7:**
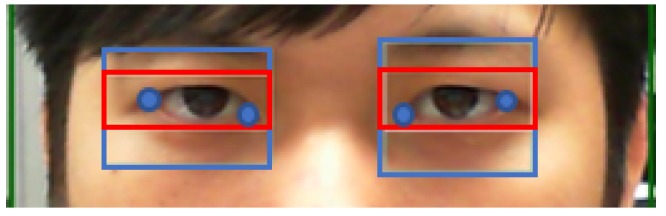
The position of the corners of the eye.

After estimating the corner of the eye, the contour of the eye can be obtained. This study takes the leftmost and rightmost points of each eye as the corners of both eyes. Figure 7 shows the result of projecting the original image after finding the corners of the eyes.

### 3.5. Feature Extraction

This study uses two features: the pupil center-eye corner vector, and the proposed method, called the inner corner-pupil center vector.

#### 3.5.1. Pupil Center-Eye Corner Vector

**Figure 8:**
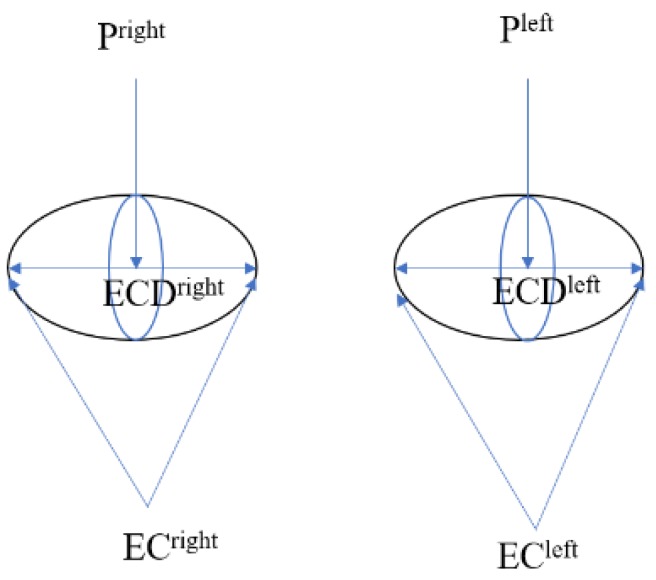
Pupil center-eye corner vector [20].

The pupil center-eye corner vector (Equation (3)) point of gaze detection algorithm is as follows:

(3) $$\left(\begin{array}{c} P_{o}R_{x}\\ P_{o}R_{y} \end{array}\right)=C\left(\begin{array}{c} 1\\ \vartheta_{N_{x}}\\ \vartheta_{N_{y}}\\ \vartheta_{N_{x}}^{2}\\ \vartheta_{N_{y}}^{2}\\ \vartheta_{N_{x}}\vartheta_{N_{y}} \end{array}\right),$$

where $P_{o}R_{x}$ and $P_{o}R_{y}$ represent the point of regard *x* and *y* coordinates ${\left(1,\vartheta_{N_{x}},\vartheta_{N_{y}},\vartheta_{N_{x}}^{2},\vartheta_{N_{y}}^{2},\vartheta_{N_{x}}\vartheta_{N_{y}}\right)}^{T}$ of feature vectors. *C* is a gazing feature coefficient matrix. The eigenvector is the Pupil Center-Eye Corner Vector $\left(\vartheta_{N}\right)$. Using the quadratic equation transformation, the following equation is defined as:

(4) $$\vartheta_{N0_{i}^{eye}}=\frac{PC^{eye}-EC_{i}^{eye}}{ECD^{eye}},$$

where $PC^{eye}$ is Equation (4) of the pupil center coordinates. eye∈{left,right} is the left eye or right eye corner coordinates. $EC_{i}^{eye}$ is the inner or outer corner of the eye. The Euclidean distance is $ECD^{eye}$, as shown in Figure 8. Equation (5) $\vartheta_{N0_{inner}^{eye}}$ and $\vartheta_{N0_{outer}^{eye}}$ is obtained and substituted into Equation (6) $\vartheta_{N1^{left}}$ and $\vartheta_{N1^{right}}$; thereby, $\vartheta_{N2}$ is obtained:

(5) $$\vartheta_{N1^{eye}}=\frac{1}{2}\left(\vartheta_{N0_{inner}^{eye}}+\vartheta_{N0_{outer}^{eye}}\right),$$

(6) $$\vartheta_{N2}=\frac{1}{2}\left(\vartheta_{N1^{left}}+\vartheta_{N1^{right}}\right).$$

This study uses $\vartheta_{N2}$ instead of $\vartheta_{N}$, that is $\vartheta_{N}=\vartheta_{N2}$. Eigenvectors ${\left(1,\vartheta_{N_{x}},\vartheta_{N_{y}},\vartheta_{N_{x}}^{2},\vartheta_{N_{y}}^{2},\vartheta_{N_{x}}\vartheta_{N_{y}}\right)}^{T}$ are used as the feature vector.

#### 3.5.2. Inner Corner-Pupil Center Vector

**Figure 9:**
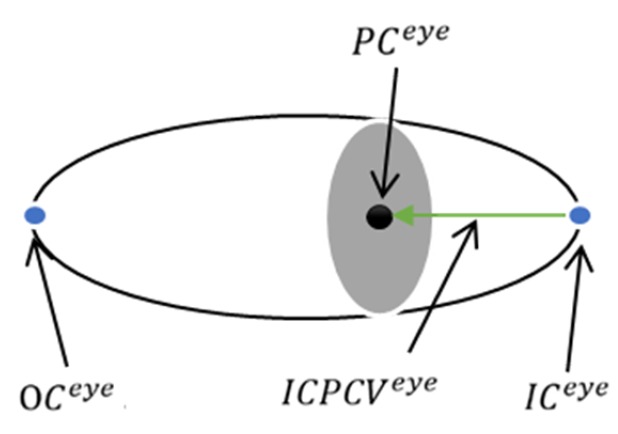
The inner corner-pupil center vector.

Inner corner-pupil center vector defines the inner corner of the eye and the center of the pupil center vector, and $ICPCV^{eye}$ represents the mathematical expression of Equation (7):

(7) $$ICPCV^{eye}=PC^{eye}-IC^{eye},$$

where $PC^{eye}$ is the pupil center position and $IC^{eye}$ is the position of the inner corner of the eye, as shown in Figure 9.

**Figure 10:**
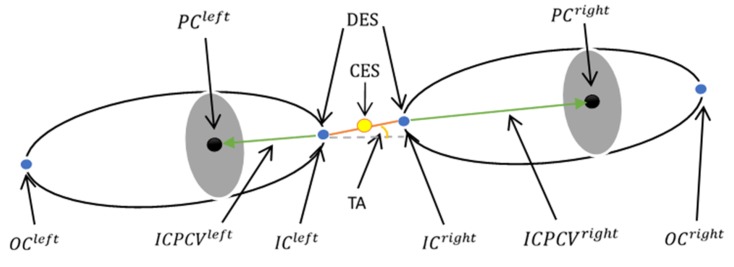
Other features.

To calculate the inner corner-pupil center vector features, this study defines several notations, as shown in Figure 10. The CES is the average position of the center of the two inner corners, and the DES is the distance between the two inner corners, while the TA is the angle between the vector and the horizontal.

Combining the above features yields the ${\left({\mathrm{CES}}_{x},{\mathrm{CES}}_{y},\mathrm{DES},\mathrm{TA},ICPCV_{x}^{left},ICPCV_{y}^{left},ICPCV_{x}^{right},ICPCV_{y}^{right}\right)}^{T}$ feature vectors.

### 3.6. Deep Neural Network

Deep neural networks (DNN) is from the neural network, but its hidden layer must be at least five layers. It is similar to the multi-layer neural network and the difference is as follows:

1. The DNN is focused on the neural network’s deep structure.
2. The features are transformed into other feature spaces between the hidden layers and it can help the prediction accuracy.

Our proposed system has 5 layers structure and the learning optimize method is Adamoptimizer. The cost function is the mean square error (MSE). The activation function of the hidden layer is rectified linear unit (ReLU) and the activation function of the output layer is sigmoid function.

## 4. Experimental Results

The features were adopted as pupil center-eye corner vector (PCECV) and inner corner-pupil center vector (ICPCV). These features are the input of DNN or multi-layer perceptron (MLP). It is important to extract the features as input because it can improve the performance without large data size. The YOLO [21] algorithm was adopted to detect the eyes to test the performance without the crafted features. The YOLO experiment was 10 users and each user was captured the 20 images. The 15 images were used as the training sample and the other five images were used as the testing sample. The average correct rate of training was 80% and the average correct rate of testing was 60%. The YOLO result is not better than our crafted features result, because it is hard to collect enough data size to tune the YOLO architecture. In addition, we provide various experiments as described in the following subsections. In all experiments, the unit of average error is the pixel.

### 4.1. Multilayer Perceptron Experiment Results

The performance of the proposed system was tested using a MLP. The MLP set the learning rate at 0.4, with 10,000 training iterations, and one hidden layer. This study tested two datasets, including a fixed head position dataset, and one in which the user’s head was free to move, using different hidden neuron numbers and different input feature vectors, as shown in Table 1 and Table 2.

### 4.2. Radial Basis Function Network Experiment Results

This study also tested the proposed method using a radial basis function network (RBFN). An RBFN is a single-layer hidden layer architecture, using different neurons. The same two data sets used for the MLP test were used for the RBFN test, and the results are shown in Table 3 and Table 4.

### 4.3. Deep Neural Network Experiment Results

The proposed system was then tested using a DNN, with the DNN learning rate set to 0.01, and 100 training iterations, while the cost function was the MSE, and the optimizer was AdamOptimizer. This study also set five hidden layers, and used the fixed head position and free head movement datasets with different numbers of hidden layer neurons to test the DNN performance, as shown in Table 5 and Table 6.

### 4.4. Eye Tracking Experiment

**Figure 11:**
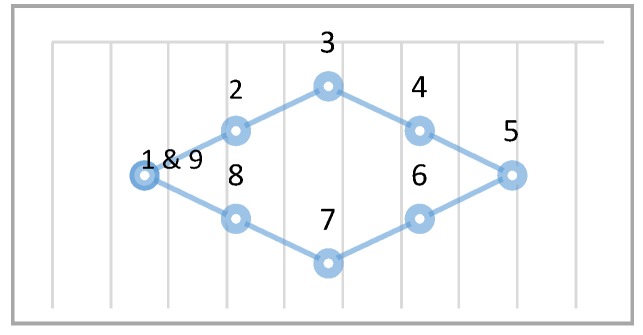
Eye movement trajectories experiment.

**Figure 12:**
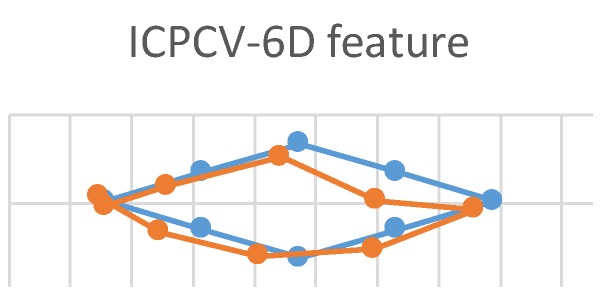
The trajectory of the head movement by MLP of inner corner-pupil center vector (ICPCV)-6D.

**Figure 13:**
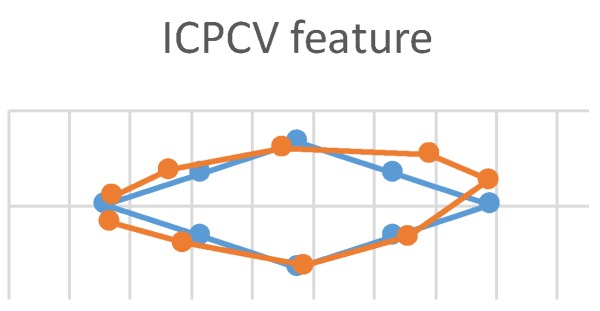
The trajectory of the head movement by MLP of ICPCV.

**Figure 14:**
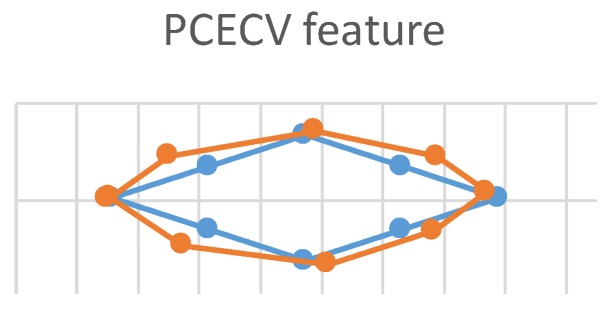
The trajectory of the head movement by MLP of pupil center-eye corner vector (PCECV).

This experiment used three feature vectors (ICPCV-6D, ICPCV, and PCECV) as MLP feature vectors to track the trajectory of the eyes. This eye movement experiment is shown in Figure 11. The users started from the leftmost point, and focused sequentially on consecutive points, moving to the right in a diamond-shaped trajectory. When the test point appeared on the screen, the users focused on the point for 10 frames. These 10 frames were then used to estimate the gaze point of an average position. The ICPCV-6D, ICPCV and PCECV feature vectors were used to test the MLP performance. Figure 12, Figure 13 and Figure 14 show the results of the head movement trajectory by MLP. Table 7 shows each average error distance between the gaze point and the actual point. From Table 7, the average error of the method using the ICPCV feature combined with the MLP is the lowest using the free head movement dataset. This experiment shows that the testers can move their heads as they wish, and we also sample the 10 frames to calculate the average distance of the focus point. Therefore, we calculated the average error distance between the gaze point and actual point, and then the overall average is calculated from three experiments. In addition, to test the DNN performance, we did the fixed-head experiments to compare the different DNN structures, as shown in the following Table 8, Table 9, Table 10, Table 11, Table 12 and Table 13.

**Figure 15:**
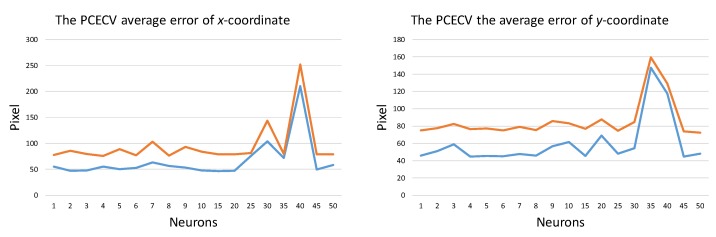
The average error of the PCECV features.

For testing PCECV performance, we calculated the average error of *x*- and *y*-coordinate. The fixed-head experiment is tested by the PCECV features with different layers (MLP), as shown in Figure 15.

For testing the head movement, the following experiments were evaluated to test the DNN performance of PCECV features, as shown in Table 14 and Table 15.

From Table 14, Table 15, Table 16, Table 17, Table 18 and Table 19, we can make some head movement discussions to compare the three features with the DNN model. If the ICPCV-6D feature with DNN is adopted, the performance is not better than the PCECV or ICPCV features. However, compared with the PCECV and ICPCV-6D functions, the performance of the ICPCV features with DNN can improve the system accuracy of *x*-*y* coordinates.

Our eye detected method was first adopted the Haar feature-based cascade classifier algorithm to detect the face. Then secondly, after detecting the face, we also adopted the Haar feature-based cascade classifier algorithm to detect the eye region. Thirdly, the image processing morphology was adopted to detect the pupil of the eye. The image processing morphology was proposed to binarized the image, and then we adopted the connected component method to find the maximum region that is the pupil of the eye. After finding the pupil of the eye, the canny method was adopted to detect the corner of the eyes. The experiment to detect eye existing or not was done as the following Table 20. We tested the 10 users and each user was captured the 9 images and their angle of the face is between $-10^{\circ }∼10^{\circ }$, as shown in Table 20. From this experiment, we can find our system to detect the eyes perfectly between $-2.5^{\circ }$ and $2.5^{\circ }$.

We compared our system with other references based on the eye-tracking user system and we described three factors based on the desktop under different operating situations, as shown in Table 21.

## 5. Conclusions

The system proposed in this study allows user head movement during the eye-tracking process. The inner corner-pupil center vector feature vectors were combined with neural networks (MLP, DNN) to improve accuracy. By so doing, the neural model is not only able to more accurately estimate the fixation point, but also allows for free movement of a user’s head, making the fixation point more accessible to the target.

Future work will use more different types of correction point numbers and distributions, collecting a greater number of different head positions or gaze angle data to train the neural network model. In addition, the model estimation accuracy can be improved in order to reduce the error caused by a change of light source, which results in unstable feature points. Future work will also explore the possibility of running eye-tracking on tablets and phones. We will test our system by the functional neuromusculoskeletal and movement-related functions/structures because the disabled users cannot easily control the computer using the mouse. Therefore, the future eye-tracking system will assist most disabled users to operate the computer.

## Figures and Tables

**Table 1:** The fixed head position data set by the multi-layer perceptron (MLP).

Feature	Coordinate	Number of Neurons	Training Average Error	Test Average Error
*ICPCV-6D*	*x*	3	47.34	59.39
	*y*	9	52.87	78.43
*ICPCV*	*x*	6	33.61	41.43
	*y*	25	41.82	58.42
*PCECV*	*x*	4	55.43	75.83
	*y*	50	48.17	72.37

**Table 2:** Free head movement data set by the MLP.

Feature	Coordinate	Number of Neurons	Training Average Error	Test Average Error
*ICPCV-6D*	*x*	5	77.86	75.81
	*y*	3	49.95	70.21
*ICPCV*	*x*	15	70.15	60.47
	*y*	8	29.88	48.94
*PCECV*	*x*	9	55.74	65.96
	*y*	6	39.91	49.58

**Table 3:** The fixed head position dataset experiment result by the radial basis function network (RBFN).

Feature	Coordinate	Number of Neurons	Training Average Error	Test Average Error
*ICPCV-6D*	*x*	10	47.66	103.20
	*y*	2	72.45	94.40
*ICPCV*	*x*	2	59.90	57.12
	*y*	2	62.11	78.93
*PCECV*	*x*	5	50.81	77.44
	*y*	10	41.25	73.31

**Table 4:** The free head movement dataset experiment result by the RBFN.

Feature	Coordinate	Number of Neurons	Training Average Error	Test Average Error
*ICPCV-6D*	*x*	7	79.99	95.42
	*y*	9	62.40	84.23
*ICPCV*	*x*	7	53.54	68.60
	*y*	15	37.02	53.92
*PCECV*	*x*	10	45.24	66.26
	*y*	5	44.14	50.46

**Table 5:** The fixed head position dataset experiment result of the deep neural network (DNN).

Feature	Coordinate	Number of Neurons	Training Average Error	Test Average Error
*ICPCV-6D*	*x*	10,20,20,20,10	25.81	43.28
	*y*	5,10,10,10,5	12.96	104.66
*ICPCV*	*x*	5,10,10,10,5	5.27	41.33
	*y*	5,5,5,5,5	20.02	63.65
*PCECV*	*x*	10,20,20,20,10	25.81	43.28
	*y*	5,10,10,10,5	12.96	104.66

**Table 6:** The free head movement dataset experiment result of the DNN.

Feature	Coordinate	Number of Neurons	Training Average Error	Test Average Error
*ICPCV-6D*	*x*	5,5,5,5,5	68.57	79.98
	*y*	5,10,10,10,5	35.56	60.35
*ICPCV*	*x*	10,20,20,20,10	11.39	54.71
	*y*	10,20,20,20,10	15.01	51.76
*PCECV*	*x*	5,10,10,10,5	20.60	57.41
	*y*	5,5,5,5,5	18.29	50.16

**Table 7:** The average error distance of the eye movement trajectory using each feature of the head movement model.

	ICPCV-6D	ICPCV	PCECV
Average error distance of experiment 1	105.92	81.65	75.21
Average error distance of experiment 2	106.64	84.38	102.45
Average error distance of experiment 3	124.40	66.36	110.13
Average of the average error distance of 3 experiments	112.32	77.46	95.93

**Table 8:** The DNN average error of *x*-coordinate in ICPCV-6D features.

Number of Neurons	5,5,5,5,5	5,10,10,10,5	10,20,20,20,10
Training average error	18.39	8.34	25.81
Testing average error	67.66	44.06	43.28

**Table 9:** The DNN average error of *y*-coordinate in ICPCV-6D features.

Number of Neurons	5,5,5,5,5	5,10,10,10,5	10,20,20,20,10
Training average error	15.46	12.96	25.12
Testing average error	118.38	104.66	127.54

**Table 10:** The DNN average error of *x*-coordinate in ICPCV features.

Number of Neurons	5,5,5,5,5	5,10,10,10,5	10,20,20,20,10
Training average error	59.26	5.27	14.10
Testing average error	63.50	41.33	41.41

**Table 11:** The DNN average error of *y*-coordinate in ICPCV features.

Number of Neurons	5,5,5,5,5	5,10,10,10,5	10,20,20,20,10
Training average error	20.02	13.97	12.75
Testing average error	63.65	64.92	67.92

**Table 12:** The DNN average error of *x*-coordinate in PCECV features.

Number of Neurons	5,5,5,5,5	5,10,10,10,5	10,20,20,20,10
Training average error	11.38	12.17	43.76
Testing average error	62.29	62.75	82.22

**Table 13:** The DNN average error of *y*-coordinate in PCECV features.

Number of Neurons	5,5,5,5,5	5,10,10,10,5	10,20,20,20,10
Training average error	7.53	18.05	8.38
Testing average error	68.23	74.24	69.97

**Table 14:** The head movement of *x*-coordinate dataset using DNN of PCECV features.

Number of Neurons	5,5,5,5,5	5,10,10,10,5	10,20,20,20,10
Training average error	37.56	25.47	36.12
Testing average error	62.93	65.18	70.22

**Table 15:** The head movement of *y*-coordinate dataset using DNN of PCECV features.

Number of Neurons	5,5,5,5,5	5,10,10,10,5	10,20,20,20,10
Training average error	42.62	37.58	50.11
Testing average error	65.17	57.81	55.71

**Table 16:** The head movement of *x*-coordinate dataset using DNN of ICPCV-6D features.

Number of Neurons	5,5,5,5,5	5,10,10,10,5	10,20,20,20,10
Training average error	62.38	36.25	58.16
Testing average error	73.16	80.40	84.21

**Table 17:** The head movement of *y*-coordinate dataset using DNN of ICPCV-6D features.

Number of Neurons	5,5,5,5,5	5,10,10,10,5	10,20,20,20,10
Training average error	74.01	35.56	38.93
Testing average error	71.89	60.35	79.16

**Table 18:** The head movement of *x*-coordinate dataset using DNN of ICPCV features.

Number of Neurons	5,5,5,5,5	5,10,10,10,5	10,20,20,20,10
Training average error	26.09	15.15	11.39
Testing average error	55.21	60.40	54.71

**Table 19:** The head movement of *y*-coordinate dataset using DNN of ICPCV features.

Number of Neurons	5,5,5,5,5	5,10,10,10,5	10,20,20,20,10
Training average error	26.65	9.72	15.01
Testing average error	53.77	53.78	51.76

**Table 20:** The effects of different angles.

	$-10^{\circ }$	$-7.5^{\circ }$	$-5^{\circ }$	$-2.5^{\circ }$	$0^{\circ }$	$2.5^{\circ }$	$5^{\circ }$	$7.5^{\circ }$	$10^{\circ }$
User 1	×	◯	◯	◯	◯	◯	◯	×	◯
User 2	×	◯	◯	◯	◯	◯	×	◯	×
User 3	◯	×	◯	◯	◯	◯	◯	◯	×
User 4	◯	◯	◯	◯	◯	◯	◯	×	◯
User 5	×	×	◯	◯	◯	◯	◯	◯	×
User 6	×	◯	◯	◯	◯	◯	◯	◯	◯
User 7	×	◯	◯	◯	◯	◯	×	×	×
User 8	◯	×	×	◯	◯	◯	◯	◯	×
User 9	◯	◯	◯	◯	◯	◯	◯	×	×
User 10	×	◯	◯	◯	◯	◯	◯	◯	◯
	40%	70%	90%	100%	100%	100%	80%	60%	40%

**Table 21:** Comparison with other reference systems.

Paper Reference	Setup (Camera, LED)	Accuracy/Metrics	Operating Condition
[22]	Commercial tracker, 1 camera	61.1%	User dependent
[23]	Commercial tracker, 1 camera	Error rate 15%	None
[24]	Commercial tracker, 1 camera	Completion time, no. of hits/misses	None
[25]	1 camera	Mean error rate 22.5%	None
Our system	1 camera	100% ($-2.5^{\circ }∼2.5^{\circ }$)	None
